# Cases of Vesical Calculi

**Published:** 1886-09

**Authors:** Charles F. Pickering

**Affiliations:** Surgeon to the Bristol General Hospital


### CASES OF VESICAL CALCULI. By Charles F. Pickering, F.R.C.S., Surgeon to the Bristol General Hospital.

*Case I.* J. M., a very badly-nourished and puny child, aged 18 months, was admitted on May 26th into the hospital, suffering from acute retention of urine. The mother's account was, that it had never been strong, had suffered from no trouble with the water before, and never had passed blood nor complained of pain in micturition. Four days before admission she noticed the child seemed in great pain, and took it to a doctor, who said it had stoppage of the bowels. The child continued to get worse, and for the last two days had not passed any water. On admission, the bladder was found distended up to the umbilicus. A catheter was passed, and six ounces of water drawn off, which was of foul odour and deposited one-third pus (?) on standing. The house-surgeon passed a sound, and thought he felt a stone once, but not again. I saw the child next day; but found it so ill, that I did not care to sound again. Morning temperature, 102°. On the following day, when I next saw the child, it was *in articulo mortis.* I examined with a sound, without chloroform, but could not detect any stone. The child died a few hours afterwards. The temperature had risen continuously ever since admission to the time of death, and the child had passed no water except by the catheter. The only sign of stone in this case was the retention of urine. I did not examine by the rectum, as at no time whilst in the hospital did the condition of the child allow of a cutting operation. The death was probably due to a condition which is called, for want of a better name, "urethral fever."

*Post-mortem Examination.*—The kidneys were somewhat enlarged and swollen, and the pelvis of each kidney a good deal dilated; in other respects these organs were apparently healthy. The bladder was slightly inflamed, and contained, lying free in its cavity, a curious specimen of vesical calculus, about the size of a bantam's egg, and of a kind that I have not seen described before.

The specimen was one of a hard calculus, enclosed in a thick fibrinous envelope. The whole weighed $3\frac{1}{3}$ drachms, and measured two inches in its long diameter and one inch in its shortest. The hard stone in the centre was the size of a pea, and was composed of uric acid. The fibrinous envelope was built up in layers of membrane, easily separated from each other, and forming a tough and compact shell. Fibrinous calculi in the bladder are occasionally seen; but this specimen has, I think, a different pathological origin from fibrinous calculi in general, which are usually changed blood-clots. It appears to me that the formation of the thick membrane in this case is best explained by the German view of stone formation: that a calculus-forming catarrh is set up on the entrance of a stone or foreign body into the bladder, which gives rise to the production of "fibrinous mucus," and this forms the adhesive material which binds together the inorganic salts. In this specimen the adhesive material was deposited, but the salts are absent in the outer layers, and thus prevented the stone being felt by the sound.

*Case II.* A young man, aged 16, was admitted into the hospital, complaining of incontinence of urine. On examination I found a very hard calculus, which I thought was oxalic acid in composition. This proved to be the case, as when, a few days after admission, I proceeded to perform litholapaxy, the stone proved too hard for the lithotrite, and the instrument gave way. I determined, as the patient was young, to remove the stone immediately by the lateral operation. This I did, and the patient left the hospital well in fourteen days. I have cut seven times for stone successfuly—once by the median, five times by the lateral, and once by the supra-pubic method. With regard to the lateral, it is becoming the practice to depreciate this time-honoured and successful operation, and to put in its place for all cases the supra-pubic operation. This, I think, is a swing of the pendulum too far in the opposite direction; for whilst I believe that the lateral is the best for children, with regard to adults the case is different, and here the supra-pubic operation should be performed; and I have no doubt it will prove, in these cases, to be the safest. I see Sir H. Thompson discards antiseptics, and does not sew up the wound in the bladder. I should prefer to perform the operation under strict antiseptic precautions, accurately suture the wound in the bladder, and drain the bladder antiseptically; this latter I found the most difficult part of the treatment, as blood-clots and mucus are very liable to frequently block the opening in the catheter, thus necessitating frequent change of the instrument. By constant care and forethought these difficulties may be overcome.

